# Identification of Prominin‐2 as a new player of cardiomyocyte senescence in the aging heart

**DOI:** 10.1111/acel.14204

**Published:** 2024-05-17

**Authors:** D. Maggiorani, Y. Santin, K. Formoso, E. Drapé, H. Martini, S. Brun, G. Cousin, O. Lairez, F. Lezoualc'h, A. Parini, V. Douin‐Echinard, J. Mialet‐Perez

**Affiliations:** ^1^ Institute of Metabolic and Cardiovascular Diseases (I2MC), UMR‐1297 INSERM, University of Toulouse Toulouse France; ^2^ Rangueil Hospital, CHU Toulouse France; ^3^ RESTORE Research Center, UMR‐1301, INSERM, CNRS, EFS University of Toulouse Toulouse France; ^4^ Univ Angers, INSERM, CNRS, MITOVASC, Equipe MitoLab, SFR ICAT Angers France

**Keywords:** aging, cardiomyocyte, heart, senescence

## Abstract

The aging heart is characterized by a number of structural changes leading to ventricular stiffness, impaired resistance to stress and increased risk of developing heart failure (HF). Genetic or pharmacological removal of senescent cells has recently demonstrated the possibility to relieve some cardiac aging features such as hypertrophy and fibrosis. However, the contribution of the different cell types in cardiac aging remains fragmentary due to a lack of cell‐specific markers. Cardiomyocytes undergo post‐mitotic senescence in response to telomere damage, characterized by persistent DNA damage response and expression of the classical senescence markers p21 and p16, which are shared by many other cell types. In the present study, we used transcriptomic approaches to discover new markers specific for cardiomyocyte senescence. We identified Prominin2 (Prom2), encoding a transmembrane glycoprotein, as the most upregulated gene in cardiomyocytes of aged mice compared to young mice. We showed that Prom2 was upregulated by a p53‐dependent pathway in stress‐induced premature senescence. Prom2 expression correlated with cardiomyocyte hypertrophy in the hearts of aged mice and was increased in atrial samples of patients with HF with preserved ejection fraction. Consistently, Prom2 overexpression was sufficient to drive senescence, hypertrophy and resistance to cytotoxic stress while Prom2 shRNA silencing inhibited these features in doxorubicin‐treated cardiac cells. In conclusion, we identified Prom2 as a new player of cardiac aging, linking cardiomyocyte hypertrophy to senescence. These results could provide a better understanding and targeting of cell‐type specific senescence in age‐associated cardiac diseases.

## Graphical abstract

Aged cardiomyocytes specifically express a transmembrane glycoprotein Prominin2 (Prom2), with unknown function in the heart. Prom2 expression correlates with cardiomyocyte hypertrophy in hearts of aged mice and is increased in atrial samples of old patients with heart failure with preserved ejection fraction (HFpEF). Prom2 expression can be regulated by a p53‐dependent signaling pathway. In vitro, Prom2 overexpression is sufficient to drive DNA damage, senescence, SASP and resistance to cell death while Prom2 shRNA silencing inhibits these features.

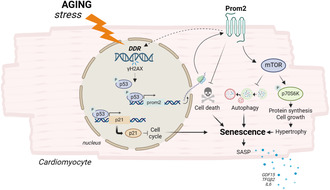

**Abbreviations**
BafA1: Bafilomycin A1
Bcl2: BCL2 apoptosis regulator
CCR2: C‐C chemokine receptor type 2
CDKN: Cyclin Dependent Kinase Inhibitor
Cyp2b10: cytochrome P450, family 2, subfamily b, polypeptide 10
DCM: dilated cardiomyopathy
DDR: DNA damage response
Doxo: doxorubicin
Fok1: endonuclease Fok1
FS: fractional shortening
GAPDH: glyceraldehyde‐3‐phosphate dehydrogenase
GDF15: growth differentiation factor 15
H_2_O_2_
: hydrogen peroxide
HF: heart failure
HFpEF: heart failure with preserved ejection fraction
HFrEF: heart failure with reduced ejection fraction
HSF1: heat shock transcription factor 1
HW: heart weight
ICM: ischemic cardiomyopathy
IL‐1ß: interleukin‐1β
IL‐6: interleukin‐6
IVST: interventricular septum thickness
Kcnk1: potassium two pore domain channel subfamily K member 1
LVEF: left‐ventricular ejection fraction
MAO‐A: monoamine oxidase‐A
MDM2: MDM2 Proto‐Oncogene
mTOR: mechanistic target of rapamycin kinase
MVBs: multivesicular bodies
Pah: phenylalanine hydroxylase
Prom2: prominin2
ROS: reactive oxygen species
RPLP0: ribosomal protein lateral stalk subunit P0
S6K: S6 kinase
SASP: senescence‐associated secretory phenotype
SA‐βGal: senescence associated β‐galactosidase
SIPS: stress‐induced premature senescence
TAF: telomere associated foci
TGFβ2: transforming growth factor β2
TL: tibia lenght
TRF1: telomeric repeat binding factor 1
TYR: tyramine
WGA: wheat germ agglutinin
β2M: β2 microglobulin
γH2AX: phosphorylated Histone H2AX

## 1 INTRODUCTION

Advanced age is accompanied by structural changes in the heart such as cardiomyocyte hypertrophy, extracellular matrix deposition, and inflammation, that contribute to diastolic dysfunction and render the elderly more prone to develop HF with preserved ejection fraction (HFpEF) (Lazzarini et al., 2013). Among these changes, accumulation of senescent cells is strongly associated with cardiovascular aging (Owens et al., 2021). Cellular senescence is characterized by a permanent cell cycle arrest with phenotypic alterations such as metabolic reprogramming, proinflammatory secretome, and maintenance of viability. Studies based on the genetic or pharmacological (senolysis) elimination of senescent cells have provided the first demonstrations of their detrimental role in cardiac aging features such as hypertrophy and fibrosis (Anderson et al., 2019; Baker et al., 2016; Lewis‐McDougall et al., 2019; Zhu et al., 2015). Growing evidence now indicates that senescent cells exacerbate the onset and progression of numerous cardiovascular disorders including post‐ischemic ventricular remodeling, angiotensin II‐induced heart failure (HF) or chemotherapy‐induced cardiotoxicity, showing that senescence pathways could be shared between aging and adverse ventricular remodeling (Demaria et al., 2017; Dookun et al., 2020; Jia et al., 2020; Owens et al., 2021; Salerno et al., 2022; Walaszczyk et al., 2019). Overall, those studies do not allow to decipher the respective contributions of the different cardiac cell lineages in age‐associated heart diseases, as the role of senescent cells was investigated systemically. Many different cardiac lineages can become senescent but the resulting effect on cardiac aging and HF remains unclear (Sun et al., 2022; Sweeney et al., 2023). Mesenchymal stromal cells have been shown to undergo replicative senescence in the aging heart with upregulation of p16 and p21 and acquisition of a senescence‐associated secretory phenotype (SASP), favoring monocyte recruitment with an increase ratio of monocyte‐derived CCR2^+^ macrophages and IL‐1ß production (Martini et al., 2019). This cascade of events is known to promote pathological cardiac remodeling also in human (Bajpai et al., 2018; Patel et al., 2018). Post‐mitotic cardiomyocytes are also prone to senescence induction. We and others demonstrated that specific telomere dysfunction, either by genetically conditioned shortening (Sahin et al., 2011) or by increased telomere‐associated foci (TAFs) during aging (Anderson et al., 2019) could activate DNA damage response associated with p53‐dependent pathway. In addition, cardiomyocyte TAFs were promoted by mitochondrial oxidative stress and resulted in activation of the classical markers of senescence (SA‐βgal, p15, p16, p21) and hypertrophy, a detrimental feature in the aging heart (Anderson et al., 2019). Furthermore, cardiomyocytes with excessive oxidative stress due to overexpression of monoamine oxidase‐A (MAO‐A) induced bystander premature senescence of cardiac stromal cells, favoring the recruitment of CCR2^+^ monocytes and the installation of cardiac inflammation (Martini et al., 2021). On the other hand, studies performed on cardiac fibroblasts have shown contrasting results, indicating that fibroblast senescence might be beneficial in some particular timing or conditions in the heart (Sawaki et al., 2018).

In light of these considerations, none of the senescence markers identified so far appear to be cell‐specific. Therefore, there is a strong need to delineate cardiomyocyte‐specific senescence pathways associated with physiological and pathological heart remodeling. In this study, we identified a network of genes (Pah, Kcnk1, Cyp2B10, and Prom2) with enhanced expression in cardiomyocytes from aged mice and we investigated their tissue and cell specificity. Despite being all highly specific for the heart, Prom2 was the only gene to be conserved in both Rat and Human. In addition, Prom2 expression increased in different models of stress‐induced premature senescence ( SIPS) through a p53‐dependent pathway. *In vitro*, Prom2 was sufficient to promote senescence, hypertrophy and resistance to cell death through Bcl‐Xl and Bcl2 upregulation, while reducing autophagic flux. Our results identify Prom2 as a new player of cardiomyocyte senescence and a potential biomarker for pathological cardiac aging.

## 2 MATERIALS AND METHODS

### 2.1 Animals and procedure

For evaluation of gene expression, male C57BL/6J mice were purchased from Janvier‐Labs and studied at the age of 3 months (young group, Y) and 20 months (old group, O). Heart and other organs (liver, kidney, lung, brain, skeletal muscle, and intestine) were collected and snap‐frozen in liquid nitrogen. Sprague–Dawley male rats from Janvier‐Labs were euthanized at 3 months (young group, Y) or 24 months old (old group, O) and the whole heart was collected and snap‐frozen in liquid nitrogen. All animals were maintained under specific pathogen‐free conditions and handled according to procedures performed in accordance with the recommendations of the European Accreditation of Laboratory Animal Care (86/609/EEC) and guidelines established by the Ethics and Animal Safety Committee of INSERM Toulouse/ENVT (agreement number: C31 555 07).

### 2.2 Echocardiography

C57Bl6 mice were anesthetized with 2% isoflurane and subjected to non‐invasive echocardiography using a Vevo2100 Visual Sonics system. Cardiac ventricular dimensions were measured in a blinded fashion on M‐MODE/2D images for the number of animals indicated.

### 2.3 Human sampling

According to the protocol n°EudraCT 2014‐000198‐38, right atrial tissues were obtained from surgical residues at the cannulation site of patients referred for cardiac surgery at the Toulouse University Hospital, Toulouse, France. Patients less than 60 years were classified as Young and patients over 60 years were classified as old, according to the W.H.O. Then, patients were stratified according to the presence or absence of cardiomyopathy (Table 1). In the group without cardiomyopathies (A and B), young or old patients were referred to surgery for valvular disease, coronary artery disease or aneurysm. In the groups with heart failure (HF) (C and D), a sub‐classification of old patients was performed according to the left ventricular ejection fraction (LVEF) by transthoracic echocardiography following the European Society of Cardiology guidelines into: (i) HF with preserved ejection fraction (HFpEF), characterized by LVEF ≥50% and left ventricular hypertrophy (interventricular septum thickness >12 mm) that were referred to surgery for aortic stenosis; (ii) HF with reduced ejection fraction (HFrEF: LVEF <50%), that were referred to surgery for ischemic cardiomyopathies (ICM) or dilated cardiomyopathies (DCM).

**TABLE 1:** Patient demographics and characteristics.

	A	B	C	D
Group	Young	Old	Old HFpEF	Old HFrEF
*n*	8	19	12	11
Baseline characteristics				
Age	47 ± 3	73 ± 1	72 ± 2	73 ± 2
Sex (male)	6/8 (75%)	14/19 (74%)	8/12 (67%)	10/11 (91%)
BMI (kg/m^2^)	26.9 ± 1.0	27.3 ± 0.8	27.9 ± 1.1	24.1 ± 0.9
Cardiac parameters				
LVEF (%)	60 ± 2	61 ± 2	60 ± 2	38 ± 3
NT‐proBNP	341 ± 104	342 ± 82	1188 ± 245	1129 ± 186.94
AF	1/8 (12.5%)	7/19 (36.8%)	4/12 (33.3%)	1/11 (9%)
Comorbidities				
Diabetes	1/8 (13%)	4/19 (21%)	5/12 (41%)	3/11 (27%)
Hypertension	2/8 (25%)	11/19 (58%)	7/12 (58%)	8/11 (73%)
Dyslipidemia	2/8 (25%)	10/19 (53%)	5/12 (42%)	6/11 (55%)
Ever‐smoker	2/8 (25%)	4/19 (21%)	4/12 (33%)	8/11 (73%)
Medications				
ß‐blockers	4/8 (50%)	13/19 (68.4%)	5/12 (41.7%)	10/11 (90.9%)
ACE inhibitor/ ARB	2/8 (25%)	5/19 (26.3%)	5/12 (41.7%)	11/11 (100%)
Calcium channel blockers	1/8 (12.5%)	2/19 (10.5%)	4/12 (33.3%)	4/11 (36.4%)
Antiplatelet	3/8 (37.5%)	12/19 (63.2%)	4/12 (33.3%)	11/11 (100%)
Anticoagulant	2/8 (25%)	7/19 (36.8%)	3/12 (25%)	3/11 (27.3%)
Diuretic	2/8 (25%)	6/19 (31.6%)	6/12 (50%)	10/11 (90.9%)
Antiarrhythmic	0/8 (0%)	3/19 (15.8%)	3/12 (25%)	1/11 (9%)

*Note*: Continuous data are presented as median (±sem) and categorical variables as frequencies (percentages).

Abbreviations: ACE, angiotensin‐converting enzyme; AF, atrial fibrillation; ARB, angiotensin receptor blocker; BMI, body mass index; LVEF, left ventricular ejection fraction.

### 2.4 Cell culture

H9C2 cells from ATCC (ATCC® CRL‐1446™) were cultivated with DMEM‐Glutamax (Gibco) containing 10% SVF (Sigma‐Aldrich) and 1% penicillin–streptomycin (Sigma‐Aldrich). Cells were incubated at 37°C under 5% CO_2_ in a humidified atmosphere. During 96 h, the cells were daily treated with a freshly prepared solution of Doxorubicin (Sigma‐Aldrich) at 100 nM or Nutlin‐3a (Sigma‐Aldrich) at 5 μM. Negative control treatments were performed with distilled water for Doxo and DMSO 0.1% for Nutlin‐3a. For plasmid transfection, an ORF (NM_144707) of Prom2 cloned in pEnter vector and tagged with C‐terminal Flag‐His was obtained from Vigene Biosciences (Charles River lab). The pCMV6‐GFP‐Prom2 fusion plasmid was from Origene Technologies (Rockville, USA). The Prom2 Rat shRNA Plasmid (Locus ID 192211) and CT Rat shRNA plasmid were from Origene Technologies (Rockville, USA).

### 2.5 Lentiviral transduction and quantification

The following lentivectors were provided by Flash Therapeutics® (Toulouse, France): rLV_pTP53RE.TagZsGreen (p53 response element [p53RE]) and rLV.pCDKN1A.TagTdTomato (p21). Transduction was performed at 80 multiplicity of infection (MOI) with 4 μg/mL of polybrene. After 6 h, the vectors were removed, cells were washed and incubated with fresh medium for 72 h. Then, cells were treated with Doxo or Nutlin‐3a, as described above. After treatment, cells were washed with PBS and fixed 10 min with 4% paraformaldehyde (PFA). After nuclei staining with DAPI (Sigma), cells were layered with mounting medium (fluoromount aqueous mounting medium, Sigma Aldrich). Image acquisition was performed on a Zeiss Axio Observer Z.1 m inverted microscope (Carl Zeiss). The cell areas of about 100 cells were quantified for each condition with imageJ software. Positive cells that displayed high levels of fluorescence in the whole cytoplasm were circled for cell surface measurement. Then, H9C2 cells with low background level of fluorescence for ZsGreen or TdTomato were revealed by manually increasing the intensity of fluorescence to measure cell surface area.

### 2.6 Adult ventricular cardiomyocytes isolation

After injection of intraperitoneal pentobarbital (200 mg/kg) and heparin (150 units), hearts were quickly excised and the aorta was cannulated for retrograde perfusion in a Langendorff apparatus at a constant flow rate of 3 mL/min at 37°C. The heart was perfused for 5 min with Krebs buffer (130 mM NaCl, 5.4 mM KCl, 1.4 mM MgCl_2_, 0.4 mM NaH_2_PO_4_, 4.2 mM HEPES, 10 mM glucose, 20 mM taurine and 10 mM creatine monohydrate, pH 7.2), followed by 8–10 min of digestion with Krebs buffer containing 12.5 μM CaCl2 (Sigma Aldrich) and 0.52 Wünsch/mL of liberase TM (Roche Diagnostics). After the digestion, the heart was removed from apparatus and the left ventricle was dissected and minced with scissors. After complete releasing of cardiomyocytes, cells were placed in a stopping buffer containing Krebs buffer, CaCl2 (12.5 μM) and 1% bovine serum albumin (Sigma‐Aldrich). Dispersed myocytes were then filtered through a 200 μm mesh (Dutscher) and allowed to sediment by gravity for 10 min. The supernatant was removed and the pellet kept for RNA extraction at −80°C.

### 2.7 Non‐cardiomyocyte cells isolations

Hearts were harvested from mice after intraventricular perfusion of 10 mL PBS, minced with scalpels, and digested twice with 0.13 Wünsch/mL of Liberase TM (Roche, Boulogne‐Billancourt, France) diluted in RPMI1640 (GIBCO, Thermo Fisher Scientific, Illkirch, France) for 10 min at 37°C. This enzymatic digestion protocol did not allow the recovery of viable cardiomyocytes. After digestion, RPMI medium +10% SVF (Sigma) was added to stop the enzymatic reaction and the cellular fraction was filtered through 100 μm‐ and 40 μm‐mesh cell strainers (Dutscher) to remove large cardiomyocyte fragments. Non‐cardiomyocyte cells were pelleted by centrifugation (9 min, 1300 rpm).

### 2.8 RNA extraction from cells and tissue

Cells were lysed with RLT from RNA easy plus mini‐kit (Qiagen) supplemented with 1% β‐mercaptoethanol. For whole tissues, 30 mg was lysed in RLT + β‐mercaptoethanol and mechanical homogenization with beads (3 min, 30 agitations/second, Precellys). Before RNA extraction, digestion was perfomed with proteinase K (Qiagen) for 10 min at 55°C. All RNA extractions were made following instructions of the RNA easy plus mini‐kit (Qiagen). An additional step of genomic DNA elimination was performed with the RNAase Free DNase set (Qiagen). After extraction, RNA concentration and quality were evaluated by spectrophotometry (ND‐100 Nanodrop, Thermofisher).

### 2.9 Real‐Time RT‐qPCR

The RNA was retrotranscribed into cDNA by the High capacity cDNA retrotranscription kit™ (Applied Biosystems). Real‐time PCR reaction was performed on a VIAA7 thermocycler system (Applied Biosystems) in 384 wells by using the SYBR‐Green reagent (Takara‐Clontech) and specific primers. Gene transcripts were normalized to Gapdh (mouse and rat samples) and to *B2M* and *RPLP0* (human samples). Relative mRNA expression compared to control group was calculated using the comparative cycle threshold (CT) method (2^− ΔΔCt^). Primers sequences are described in Table 2.

**TABLE 2:** The sequence of primers for real‐time PCR.

Species	Gene	Forward	Reverse
Mice	*Prom2*	CCTTCCCGGCAGAGTTGATAA	AATCACAGCACACACCACGT
	*Pah*	TGGATAAGCGTAGCAAGCCC	CCAGGGCACTGTGTTCTTTTC
	*p21* (*Cdkn1a*)	GCAGAATAAAAGGTGCCACAGGC	CCGAAGAGACAACGGCACACT
	*Kcnk1*	CGGAATCACGTGTTACCTGC	TGAACTTCTTCAGCTCGTGGA
	*p16V2* (*Cdkn2a*)	CCGAACTCTTTCGGTCGTACCC	CTGCTACGTGAACGTTGCCCA
	*p15* (*Cdkn2b*)	AGATCCCAACGCCCTGAAC	CAGTTGGGTTCTGCTCCGT
	*Gapdh*	AGGTTCGGTGGAACGGATTTG	TGTAGACCATGTAGTTGAGGTCA
Human	*PROM2*	TGTAGGGCCTTGTGGGGTGA	AGAGCTACCGAAGTCCTGTGG
	*B2M*	GATGAGTATGCCTGCCGTGT	TGCGGCATCTTCAAACCTCC
	*RPLP0*	TTGCTGGCCAATAAGGTGCC	AAAAGGAGGTCTTCTCGGGC
Rat	*Prom2*	CTATAGAACCCACCGGGCAAG	GGGCTCTGGAACAGAGGTTAC
	*Pah*	CGCTGCTAAGCTAGACACCTC	AGCTTGTTTCCTGCCCAAAGT
	*Kcnk1*	GCTGGAGGCCAGCAATTATG	CCATAGCCTGTGGTGGAGAG
	*p21* (*Cdkn1a*)	TGTGATATGTACCAGCCACAGG	CGAACAGACGACGGCATACT
	*p16V2* (*Cdkn2a*)	CCCCGATACAGGTGATGATG	CAGTTCGAATCTGCACCATAGG
	*p15* (*Cdkn2b*)	GACAGGTGGAGACGGTGC	GCCCATCATCATGACCTGGA
	*GDF15*	CCGACTGCATGCCAACCAGA	CCAATCGCACCTCTGGACTGA
	*TGFβ2*	TTCCCCTCCGAAACTGTCTGC	TACCCACAGAGCACCTGGGAC
	*IL6*	GTTGCCTTCTTGGGACTGATG	GAAGTCTCCTCTCCGGACTTGT
	*Gapdh*	TCTCTGCTCCTCCCTGTTCTA	TCCGATACGGCCAAATCCGTT

### 2.10 Immunofluorescence

Hearts were included in optimal cutting temperature compound (OCT) (Sigma Aldrich) and snap frozen under ice‐cold 2‐methylbutane (Sigma Aldrich). Heart frozen sections (5 μm) were fixed 10 min with 4% PFA and permeabilized with triton X‐100 0.2% for 20 min. Then, unspecific sites were blocked with PBS, 2% BSA (Sigma Aldrich) 1 h at RT. Incubation with Prom2 antibody (Abcam Ab74997) was done O/N at 4°C. After three washes with PBS‐Tween 0.025%, incubation with secondary antibody (Goat anti rabbit‐alexa fluor 488, Thermofisher A11008) and texas‐red WGA (Thermo fisher W21405) was done. Nuclei were stained with DAPI (SIGMA) and slides were mounted with mounting medium (fluoromount Aqueous mounting medium, Sigma Aldrich). Images were acquired with confocal microscope LSM780 (Carl Zeiss). For the quantification of Prom2‐positive cardiomyocytes (Prom2^+^), images were analyzed with ImageJ software and cells harboring 80% of Prom2 staining at the plasma membrane were considered as positive with a total count of 100 cardiomyocytes per heart. Cardiomyocyte area was measured with imageJ software.

H9C2 cells were fixed 10 min with 4% PFA and permeabilized with triton X‐100 0.2% for 10 min. Then, unspecific sites were blocked with PBS, 3% BSA (Sigma Aldrich) 1 h at RT. Incubation with α‐actinin antibody (BD Transduction Laboratories 612,576) or γH2AX (Millipore 05–636) was done O/N at 4°C. After three washes with PBS and incubation with secondary antibody (Goat anti‐rabbit‐alexa fluor 594), Goat anti‐mouse‐alexa fluor 594T, nuclei were stained with DAPI (Sigma‐Aldrich) and slides were mounted with mounting medium (fluoromount Aqueous mounting medium, Sigma‐Aldrich). Images were acquired with epifluorescence microscope (Zeiss). For the quantification of Prom2‐positive cardiomyocytes (Prom2^+^), images were analyzed with ImageJ software and cell area was measured.

### 2.11 Western blot

H9C2 cells were lysed using RIPA buffer (25 mM Tris, 150 mM NaCl, 1% NP‐40, 0.1% SDS) containing phosphatase (PhosStop, Roche) and protease inhibitors (Protease inhibitor cocktail, Sigma‐Aldrich). Equal amounts of protein were separated by sodium dodecyl sulfate–polyacrylamide gel electrophoresis (SDS‐PAGE) and transferred onto nitrocellulose membrane (Transblot turbo transfer system, Biorad). After 1 h of blocking with PBS‐Tween, 5% BSA, membranes were incubated overnight at 4°C with primary antibodies from Cell Signaling Technology (anti‐phospho‐p53 CST9284; anti‐β‐Actin CST4970; anti‐Bcl2 CST2870; anti‐Bcl‐XL CST2764; anti‐LC3b CST2775; anti‐phospho‐p70S6K CST9205); Abcam (anti‐Prom2 Ab74997); Abnova (anti‐p62 H00008878); Santa Cruz Biotechnology (anti‐p21 SCBT6246; anti‐p15/p16 SC377412). After three washes with PBS‐Tween 0.1%, secondary HRP‐ antibodies were added and incubated 2 h at RT. Proteins were revealed by ECL‐Prime (Biorad) and image acquired with ChemiDoc XRS+ camera (Biorad). Relative densities were quantified using the ImageLab 4.0 software (Bio‐Rad) and β‐Actin was used as loading control.

### 2.12 SA‐βgal assay

H9C2 cells were stained with Senescence β‐Galactosidase Staining Kit #9860 (Cell signaling technology) with staining solution adjusted at pH 6.0 and incubated O/N at 37°C.

### 2.13 LDH assay

For quantitative assessment of necrosis, LDH release in cell culture medium was measured using the LDH cytotoxicity assay kit according to the manufacturer's instructions (Biovision).

### 2.14 Statistical analysis

Results are expressed as mean ± SEM of biological replicates from different animals or independent cell experiments. The statistical significance between two groups of samples was estimated with the unpaired Student's two tailed *t*‐test (Student's *t*‐test). Multiple comparisons were performed with 1‐way or 2‐way ANOVA followed by Tukey's post‐hoc test, and for samples without Gaussian distribution, the Kruskal‐Wallis test with Dunn's post‐hoc test. Difference between groups were tested with GraphPad Prism Software and considered significant for *p* < 0.05.

## 3 RESULTS

### 3.1 Identification of Prominin‐2 as a new marker of cardiomyocyte post‐mitotic senescence

**FIGURE 1:**
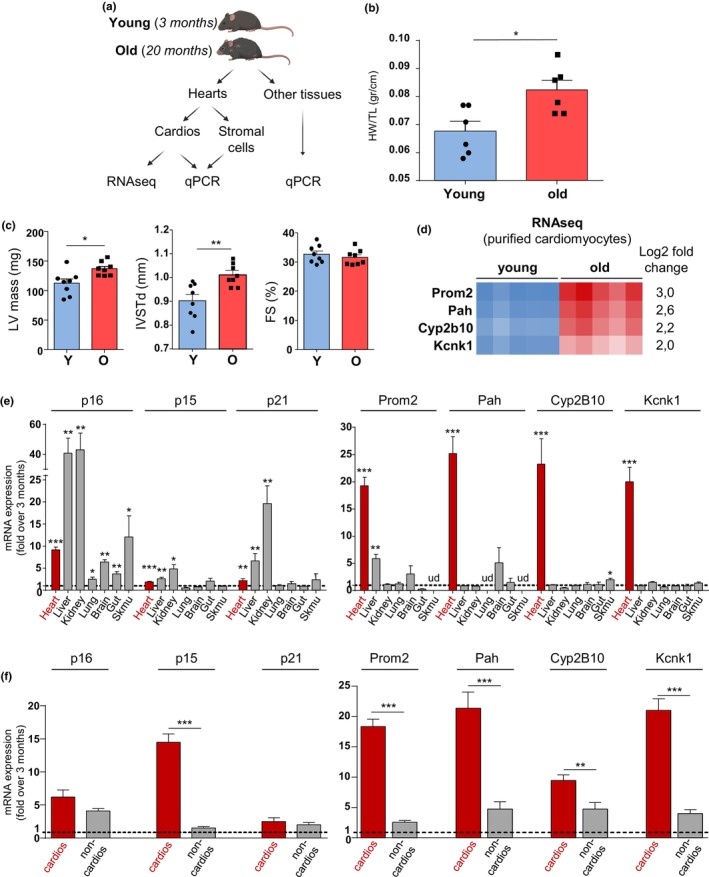
Cell‐specific and tissue‐specific expression of the cardiac senescence markers. (a) Experimental design for the isolation of tissues, cardiomyocytes (cardios) or non‐cardiomyocyte cells (non‐cardios) in young (3 months) and aged (20 months) C57Bl6J mice. (b) Heart weight (HW)‐to‐tibia length (TL) ratios in young (3 months) and aged (20 months) C57Bl6J mice. *N* = 6, *p < 0.05. (c) Echocardiographic analysis showing estimated Left Ventricle (LV) mass (mg), diastolic Interventricular Septum Thickness (IVSTd) and Fractional Shortening (FS). *N* = 8, *p < 0.05; **p < 0.01. (d) The heatmap of the most upregulated genes (log2‐fold changes) in the RNAseq analysis of purified cardiomyocytes from old mice compared to young mice (*N* = 5) (*p* < 0.001). (e) The mRNA expression of senescence markers by RT‐QPCR in seven distinct tissues of old mice (20 months) relative to young mouse tissues (3 months). Results are expressed as mean ± SEM of individual mice: heart *N* = 7, liver *N* = 6, kidney *N* = 6, lung *N* = 10, brain *N* = 4, gut *N* = 6, skeletal muscle *N* = 5. *p < 0.05; **p < 0.01; ***p < 0.001 in old vs young with Student's *t*‐test. (f) mRNA expression of senescence markers by RT‐QPCR in different cell fractions of the heart (cardiomyocytes or non‐cardiomyocytes cells) in old mice (20 months) relative to young mice (3 months). Results are expressed as mean ± SEM of individual mice: cardiomyocytes (*N* = 7) or non‐cardiomyocyte cells (*N* = 8). Statistical analysis was performed by Student's *t*‐test **p < 0.01; ***p < 0.001.

**FIGURE 2:**
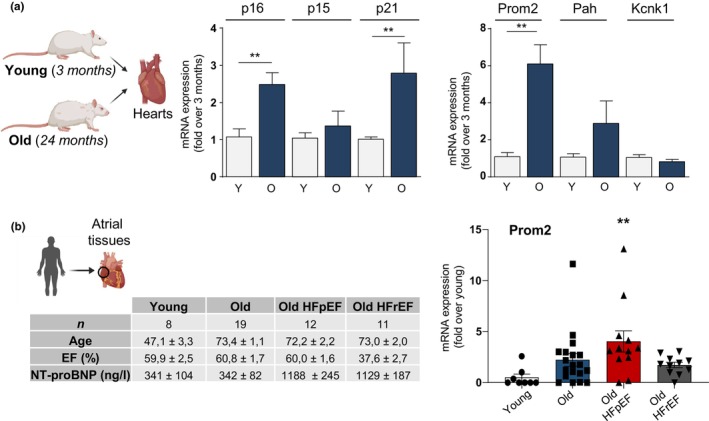
Prom2 is the most robust and conserved marker in rat and human cardiac aging. (a) mRNA expression of senescence markers by RT‐qPCR in whole hearts from young (3 months, Y) and old (24 months, O) rats. Data are expressed as mean ± SEM of relative gene expression versus Y group (*N* = 6 rats). *p < 0.05; **p < 0.01 by Student's *t*‐test. (b) mRNA expression of *PROM2* in human atrial samples of young (Y), old (O), old + Heart Failure with preserved Ejection Fraction (HFpEF) and old + HFrEF patients. Data are expressed as mean ± SEM of relative expression versus Y group. *N* = 8 Y, *N* = 20 O, *N* = 12 HFpEF, *N* = 12 HFrEF. Multiple comparison test Kruskal–Wallis test with Dunn's post‐hoc was performed test for statistical analysis *p < 0.05.

In order to delineate specific markers of cardiomyocyte senescence, we leveraged our previously published RNAseq data obtained from highly purified cardiomyocytes isolated from young and old mice (Figure 1a) (Anderson et al., 2019). Aging hearts were characterized by cardiac hypertrophy, as seen with increased HW/TL ratios, increased left ventricle mass and increased septum thickness (IVST) measured by echocardiography (Figure 1b,c). According to previous studies, we did not observed systolic dysfunction in aged mice as fractional shortening (FS %) was not modified (Abdellatif et al., 2023; Anderson et al., 2019) (Figure 1c). On the RNAseq, we selected the top four genes with the highest upregulation (Figure 1d). These genes encoded a transmembrane glycoprotein (Prom2), a potassium channel (Kcnk1), a member of the cytochrome P450 superfamily (Cyp2b10) and the phenylalanine hydroxylase (Pah). We first evaluated the cardiac specificity of Prom2, Kcnk1, Cyp2b10 and Pah by comparison with other organs such as liver, kidney, lung, brain, gut and skeletal muscle from young and old mice. As shown in Figure 1e, p16, p15 and p21 were induced at various levels in several tissues and were not specific of the aging heart, as previously reported (Krishnamurthy et al., 2004). On the other hand, aging induced specific upregulation of Prom2, Pah, Cyp2b10 and Kcnk1 in the heart, compared to other tissues (Figure 1e). In order to evaluate the cell‐specificity of these new markers, we separated cardiomyocytes from the non‐cardiomyocytes fraction, constituted by heterogeneous cardiac cell types such as endothelial cells, fibroblasts, smooth muscle cells and immune resident cells (Figure 1a). We observed a significant up‐regulation of p16 and p21 markers in both cardiomyocytes and non‐cardiomyocyte cells from old compared to young mice, whereas p15 induction was more specific for cardiomyocytes (Figure 1f). Importantly, expressions of Prom2, Pah, Cyp2b10 and Kcnk1 were strongly enriched in cardiomyocytes compared to non‐cardiomyocytes cells with aging (Figure 1f). We then translated these findings in rat and human in order to evaluate if this signature of cardiomyocyte senescence was conserved across species. In aged rat hearts (24 months), classical markers such as p16 and p21 were significantly elevated, while p15 remained unchanged (Figure 2a). Concerning the new markers, we found that only Prom2 gene expression increased, while a trend was observed for Pah (Figure 2a). No modification was observed for Kcnk1 expression and Cyp2b10 could not be measured as it is a mouse specific gene. Since Prom2 was consistently increased in aged hearts of male mice and rats, we verified that there was no sex‐biased expression with aging. In a cohort of female mice at 24 months, we also found an overexpression of Prom2 in the heart, compared to 3 months (Figure S1). Finally, in order to evaluate if Prom2 was also modified in human aged or disease hearts, we tested its expression in atrial biopsies of patients referred for cardiac surgery. While older individuals without cardiomyopathy showed a trend for increased Prom2 mRNA expression compared to young individuals, this change became significant only in the group of old individuals with HFpEF (Figure 2b), a very common aged‐related cardiac disease characterized by ventricular stiffness and impaired relaxation, together with preserved systolic function (Lazzarini et al., 2013). On the contrary, no significant upregulation of Prom2 was observed in the group of old HFrEF patients. In conclusion, our results identify Prom2 as a new cardiac‐specific transcriptional signature of aging.

### 3.2 Prom2 is associated with cardiac cell hypertrophy

**FIGURE 3:**
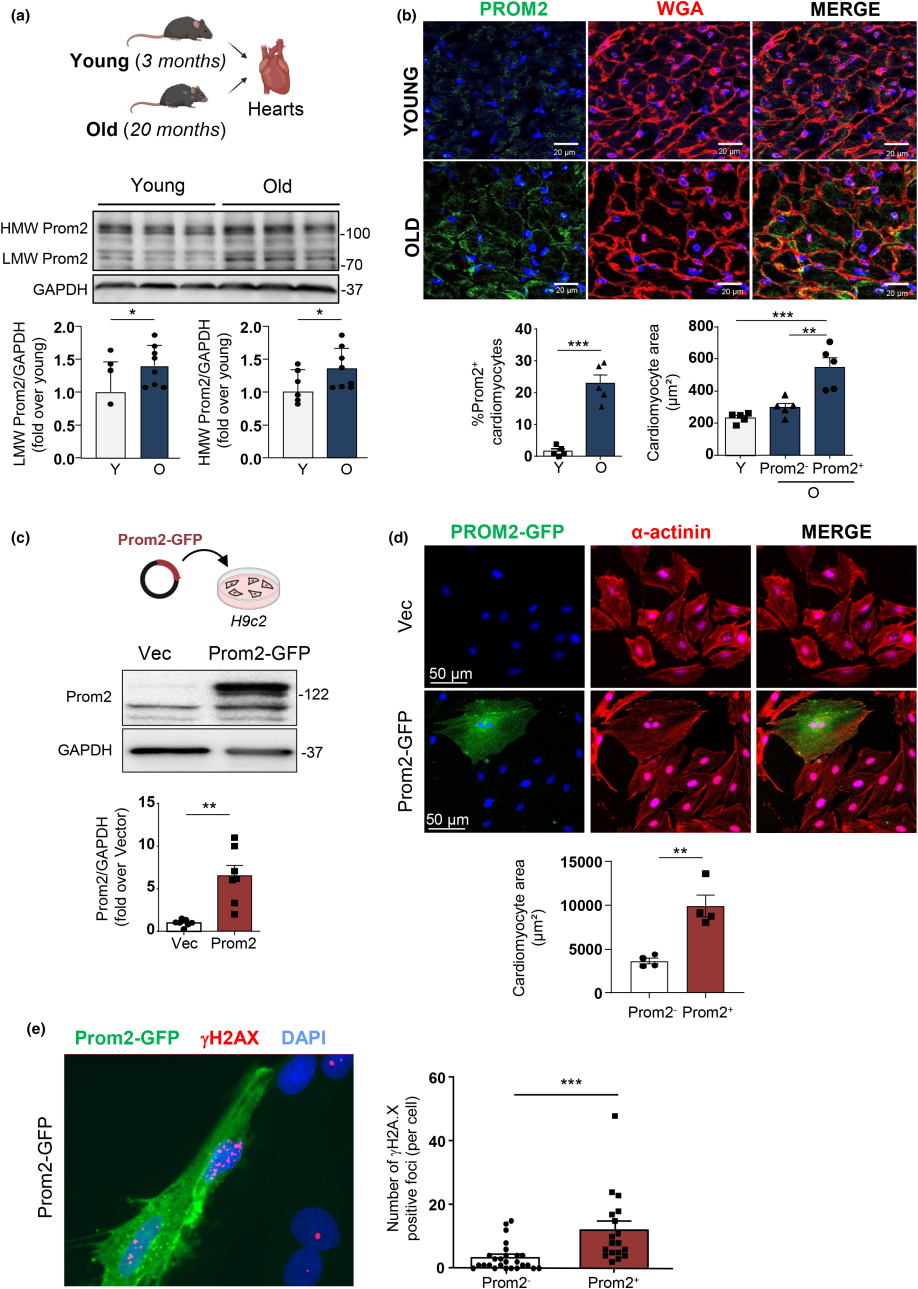
Prom2 is associated with cardiomyocyte hypertrophy in the aging heart (a) The protein levels of Prom2 by immunoblot analysis in young and old mice heart homogenates (*N* = 6–8). Upper panel: Representative immunoblots and lower panel: quantifications of Prom2 low molecular weight (LMW) and high molecular weight (HMW) normalized to GAPDH and expressed as fold over young mice. *p* < 0.05 by Student's *t*‐test. (b) Upper panel: Representative confocal acquisition of Prom2 (green) and WGA (red) immunofluorescence staining in young (3 months) and old (20 months) mice hearts. Scale bar = 20 μm. Lower left panel: Quantification of Prom2^+^ cardiomyocytes in young (Y) and old (O) mice expressed as % of total cardiomyocytes. Lower right panel: Cell area measurement of positive Prom2^+^ cardiomyocytes (green + red) or negative Prom2^−^ cardiomyocytes (Red only) in old mice compared to young mice. About 100 cardiomyocytes were manually counted and measured in each heart. Data are expressed as mean ± SEM of *N* = 6 mice per group. (c) Upper panel: Representative immunoblot expression of Prom2 and GAPDH in H9C2 cells transfected with empty vector (Vec) or Prom2‐GFP (Prom2) for 48 h. Lower panel: The quantifications of Prom2 to GAPDH expression ratio. Data are expressed as mean ± SEM of *N* = 7. (d) Upper panel: Representative fluorescent images of H9C2 cells transfected with empty Vec or Prom2‐GFP (green) for 96 h and counterstained with the cardiomyocyte marker α‐actinin (red). Bottom panel: Quantitative measurements of cell area of Prom2^+^ cells (Prom2‐GFP, green + red) compared to Prom2^−^ cells (untransfected, red only). Scale bar = 50 μm. (e) Left panel: Representative fluorescent image of H9C2 cells transfected with Prom2‐GFP (green) for 48 h and counterstained with the DNA damage marker γH2AX (red) and DAPI (Blue). Right panel: Quantitative measurements of the number of γH2AX nuclear foci in Prom2^+^ versus Prom2^−^ cells. Data are expressed as mean ± SEM of *N* = 4 experiments. Statistical analysis was performed by Student's *t*‐test or one‐way ANOVA for more than two groups. *p < 0.05; **p < 0.01; ***p < 0.001.

Prom2 is a pentaspan membrane glycoprotein related to Prominin1/CD133, a marker of stem cells, and its expression and regulation in the heart remains enigmatic (Singh et al., 2013). To evaluate Prom2 protein expression during cardiac aging, we performed immunoblotting in mouse heart homogenates (Figure 3a). Prom2 immunoblot profile revealed the presence of multiple bands characteristics of glycosylated membrane proteins (Florek et al., 2007). The apparent molecular weights of Prom2 at ~115 kDa and 90 kDa were similar to the native and N‐deglycosylated forms of Prom2 previously observed in mouse kidney membranes (Florek et al., 2007). Here, we found that both low‐ and high‐molecular weight proteins increased in aged hearts compared to the young ones (Figure 3a).

In order to better understand the distribution and localization of Prom2 during cardiac aging, we next performed immunofluorescence confocal microscopy in mouse heart cryosections. Prom2 staining was observed in cardiomyocytes of aged hearts, close to the plasma membrane, as visualized by wheat germ agglutinin co‐staining (WGA, red) whereas in young mice, Prom2 staining was very low and scattered (Figure 3b). Numeration of Prom2^+^ cardiomyocytes (green) confirmed a significant increase in aged hearts compared to young hearts (Figure 3b). In addition, we found that Prom2^+^ cardiomyocytes had significantly larger cell surface area compared to Prom2^−^ cardiomyocytes, suggesting a correlation between Prom2 expression and hypertrophy (Figure 3b). In order to establish a causal link between Prom2 and hypertrophy, we transfected H9C2 cells with a Prom2‐GFP plasmid and performed α‐actinin immunofluorescence (Figure 3c,d). Most interestingly, Prom2‐GFP‐transfected cells (Prom2^+^, red and green) displayed much larger cell area than empty vector‐transfected cells (Prom2^−^, red only), showing that Prom2 overexpression was sufficient to drive cardiac cells hypertrophy (Figure 3c,d). In addition, we observed that Prom2^+^ cells displayed higher number of nuclear foci for the DNA damage marker γH2AX compared to Prom2^−^ cells, indicating that they might be prone to DNA damage and senescence (Figure 3e).

### 3.3 Stress‐induced premature senescence enhances the expression of Prom2 in vitro

**FIGURE 4:**
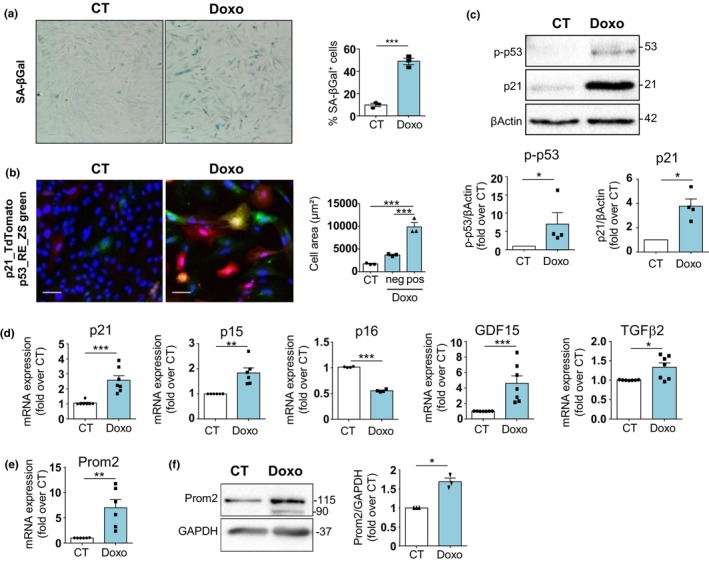
Prom2 is upregulated in stress‐induced senescence. (a) SA‐βgal activity in H9C2 cells treated or not with Doxorubicine (Doxo) for 96 h (100 nM). The number of SA‐βgal^+^ (blue) cells relative to total number of cells is displayed in the corresponding graph (right) and expressed as mean ± SEM (*N* = 3). (b) Representative fluorescence of H9C2 cells co‐transduced with the lentivirus p53RE‐ZsGreen and p21‐Tdt‐tomato, and treated or not with Doxo for 96 h (100 nM). Scale bar = 100 μm. Cell areas of positive cells for p53RE‐ZsGreen or p21‐Tdt‐tomato (pos) and negative (neg) after Doxo treatment were compared with untreated control cells (CT). Data are expressed as mean ± SEM of *N* = 4 experiments. (c) The evaluation of p21 and p‐p53 (phosphorylated p53) expressions by western blotting in Doxo‐treated cells. Data are expressed as mean ± SEM of *n* = 4 experiments. (d) mRNA expression of expression of senescence markers (p16, p15, p21) and SASP markers (GDF15, TGFβ2) by RT‐qPCR in H9C2 treated with Doxo for 96 h. Data are expressed as mean ± SEM of Doxo versus untreated CT group (*N* = 6). (e) mRNA expression of the Prom2 by RT‐qPCR in H9C2 treated with Doxo for 96 h. Data are expressed as mean ± SEM of Doxo versus untreated CT group (*N* = 6). (f) Representative immunoblot of Prom2 and GAPDH in H9C2 cells treated with Doxo (96 h). Lower panel: Quantifications of Prom2 to GAPDH expression ratio. Data are expressed as mean ± SEM of *N* = 3 experiments. Statistical analysis was performed by Student's *t*‐test (for two groups) or one‐Way ANOVA with post‐hoc tukey test (for three groups). *p < 0.05; **p < 0.01; ***p < 0.001.

Given that aging and hypertrophy are two intricately linked processes associated with cell senescence, we next assessed the role of Prom2 in different models of SIPS in vitro. We first developed a model of SIPS by treating H9C2 with Doxorubicin (Doxo) (Lerida‐Viso et al., 2022; Linders et al., 2023). As expected, Doxo‐treated H9C2 recapitulated the classical hallmarks of senescence with an increase in SA‐βgal activity (Figure 4a) and the activation of the p53 and p21 pathways, measured with a reporter gene approach (Figure 4b). For this purpose, we used lentivectors harboring reporter genes under the control of two different synthetic promoters: p53 response elements (p53RE‐ZS‐green) or p21 promoter (p21_TdT‐tomato) (Figure 4b). After lentiviral transduction, Doxo significantly increased the number of p53^+^/p21^+^ cells, which were hypertrophied compared to p53^−^/p21^−^ cells or control cells (Figure 4b). This result is consistent with our previous observations showing that senescent cardiomyocytes were hypertrophied (Manzella et al., 2018). Furthermore, activation of the p53 pathway was confirmed at the protein level, monitored by the phosphorylation of p53 protein (p‐p53) and the increase in p21 protein expression (Figure 4c). Interestingly, the upregulation of Prom2 at the mRNA and protein level was part of the Doxo‐induced senescence program, characterized by an increase in p21 and p15 gene expression, together with the previously identified cardiomyocyte SASP factors GDF15 and TGFβ2 (Anderson et al., 2019) (Figure 4d–f). We next evaluated if others SIPS triggers could promote Prom2 upregulation. For this purpose, we transfected neonatal rat cardiomyocytes with a plasmid harboring TRF1‐Fok1 endonuclease to induce specific damages to the telomeres (Dilley et al., 2016) and to promote cardiomyocyte senescence and SASP (Figure S2a). We found that TRF1‐Fok1 transfection upregulated Prom2 expression at 96 h, compared to control cells transfected with the inactive mutated form of TRF1‐Fok1D450 (Figure S2a). We next tested the mitochondrial production of reactive oxygen species (ROS) as another SISP trigger, through chronic activation of MAO‐A (Kaludercic et al., 2010, 2014; Manzella et al., 2018). We treated H9C2 cells with the MAO‐A substrate tyramine for 96 h, which was previously demonstrated to induce classical markers of senescence (Manzella et al., 2018). Again, we observed that Tyr treatment upregulated p21, p15, SASP factors GDF15 and TGFβ2, and Prom2 expression at 96 h (Figure S2b). Hence, senescent cardiac cells induced by different triggers (Doxo, TRF1‐Fok1 or MAO‐A/ROS) are all characterized by an upregulation of Prom2.

**FIGURE 5:**
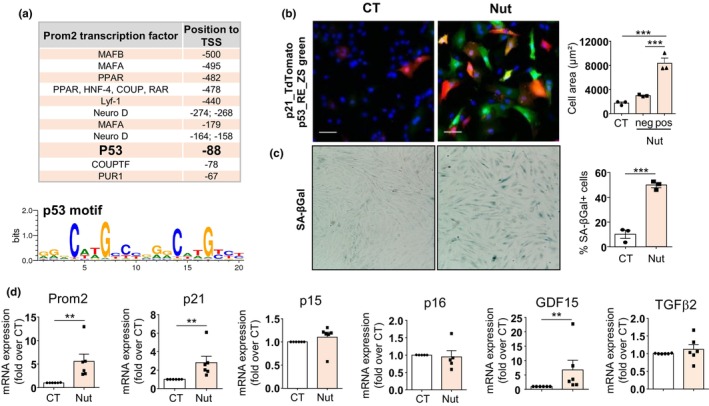
Prom2 upregulation is induced upon p53 activation. (a) The analysis of Human Prom2 promoter with “MOTIFMAP” showing putative transcription factor binding sites relative to TSS (transcription Start Site). The p53 motif present at position −88 is shown below. (b) Representative fluorescence of H9C2 cells co‐transduced with the lentivirus p53RE‐ZsGreen and p21‐Tdt‐tomato, and treated with Nutlin‐3a (Nut) for 96 h (5 μM) or DMSO 0.1% (CT). Scale bar = 100 μm. Cell area of positive cells for p53RE‐ZsGreen or p21‐Tdt‐tomato (pos) and negative (neg) after Nutlin‐3a (Nut) treatment compared with untreated control cells (CT). Data are expressed as mean ± SEM of *N* = 4 experiments. (c) SA‐βgal activity in H9C2 cells treated with Nutlin‐3a (Nut) for 96 h (5 μM) or DMSO 0.1% (CT). The number of SA‐βgal^+^ (blue) cells relative to total number of cells is displayed in the corresponding graph (right) and expressed as mean ± SEM (*N* = 3). (d) The mRNA expression of senescence markers (Prom2, p16, p15, p21) by RT‐qPCR in Nutlin‐3a‐treated cells (5 μM) for 96 h. Data are expressed as mean ± SEM of relative expression vs DMSO 0.1% CT group (*N* = 6). Statistical analysis was performed by Student's *t*‐test (for two groups) or one‐Way ANOVA with post‐hoc tukey test (for three groups). *p < 0.05; **p < 0.01; ***p < 0.001.

To better understand the underlying mechanism of Prom2 upregulation, we analyzed the putative transcription binding sites in the human Prom2 promoter and identified a p53‐specific motif at position −88 (Figure 5a). As p53 is a main trigger of senescence in the heart, we treated H9C2 cells with Nutlin‐3a, a potent inhibitor of p53‐MDM2 interaction that drives specific activation of p53. As expected, Nutlin‐3a increased the expression of the p21‐TdT‐tomato and p53‐RE ZS‐green reporter genes, as observed by the red and green staining in transduced H9C2 cells (Figure 5b) and p53^+^/p21^+^ cells were hypertrophied compared to p53^−^/p21^−^ cells or untreated cells. Nutlin‐3a treatment also increased SA‐βgal activity (Figure 5c). Most interestingly, Nutlin‐3a‐dependent SIPS induction was associated with an increase in Prom2 gene expression together with p21 and GDF15, two known target genes of p53, whereas p15 and p16 gene expression was unchanged (Baniulyte et al., 2023; Osada et al., 2007; Szak et al., 2001) (Figure 5d). Altogether, our results indicate that SIPS enhances the expression of Prom2 through a p53‐dependent mechanism.

### 3.4 Prom2 overexpression is sufficient to drive senescence markers in H9C2 cells

**FIGURE 6:**
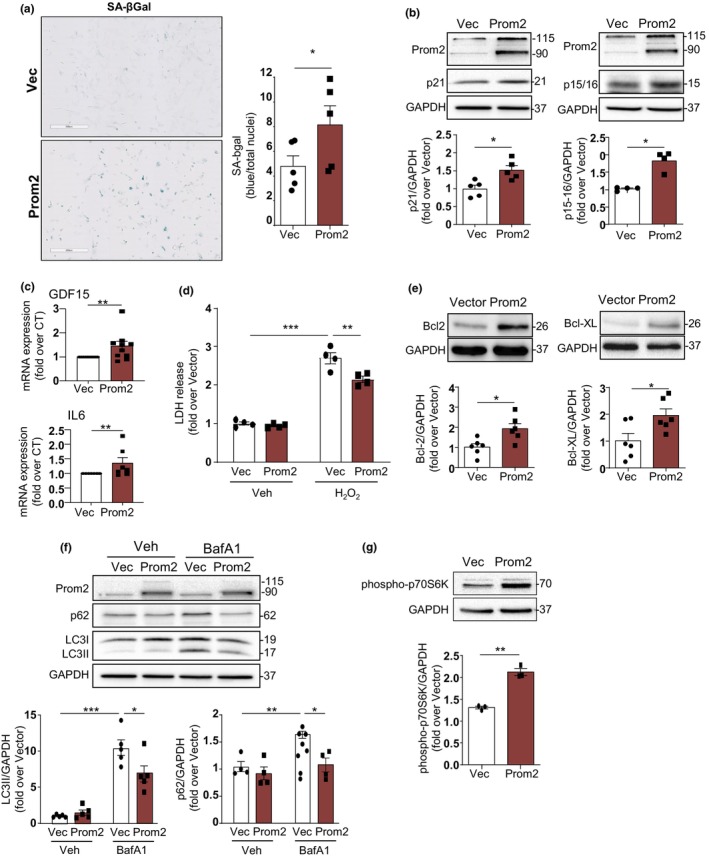
Prom2 recapitulates the main features of cell senescence. (a) Representative images of SA‐βgal activity in H9C2 cells transfected with empty vector (Vec) or pENTER‐Prom2 (Prom2) for 96 h. scale bar = 500 μm. The area of blue, indicative of SA‐βgal^+^ cells, divided by the total number of nuclei is displayed in the graph and expressed as mean ± SEM (*N* = 5). (b) Representative immunoblot and the quantitative expression of Prom2, p21 and p15/p16 proteins normalized to GAPDH in H9C2 cells transfected with empty Vec or pENTER‐Prom2 for 72 h. Data are expressed as mean ± SEM of *N* = 4–5 experiments. (c) GDF15 and IL6 mRNA expression in H9C2 cells transfected with empty Vec or pENTER‐Prom2 for 72 h. Data are expressed as mean ± SEM (*N* = 10). (d) LDH release in the supernatant of H9C2 cells transfected with empty Vec or pENTER‐Prom2 for 72 h and treated with Veh or 1 mM H_2_O_2_ for 24 h. Data are expressed as mean ± SEM (*N* = 4). (e) Representative immunoblot and quantitative expressions of Bcl2 and Bcl‐XL/GAPDH in H9C2 cells transfected with empty Vec or pENTER‐Prom2 for 72 h. Data are expressed as mean ± SEM (*N* = 6). (f) Representative immunoblot and quantitative expressions of Prom2, LC3 and p62 relative to GAPDH in H9C2 cells transfected with empty Vec or pENTER‐Prom2 for 72 h and treated with Veh or BafA1 (100 nM) for 1 h. (g) Representative immunoblot and quantitative expressions of phospho‐p70S6K to GAPDH ratios in H9C2 cells transfected with empty Vec or pENTER‐Prom2 for 72 h. Data are expressed as mean ± SEM of *N* = 3–5 experiments. Statistical analysis was performed by Student's *t*‐test or two‐way ANOVA for more than two groups. *p < 0.05; **p < 0.01; ***p < 0.001.

Next, we evaluated whether Prom2 could play a direct role in the induction of the senescent phenotype in cardiac cells. Following Prom2 plasmid transfection, we demonstrated an induction of SA‐βgal activity at 96 h (Figure 6a), together with a significant increase in p21 and p15/p16 protein expression at 72 h (Figure 6b). SASP gene expression by RT‐PCR also indicated an increase in the expression of GDF15 and IL6 in Prom2‐transfected cells (Figure 6c) while TGFβ2 was unchanged (not shown). As senescent cells are known to be resistant to cell death, we evaluated if the increase of Prom2 could activate some specific cell survival signaling pathways. As shown in Figure 6d, Prom2 overexpression enhanced cardiac cell survival in the presence of a cytotoxic dose of H_2_O_2_ (Kwon et al., 2003). Accordingly, expression levels of the cell survival proteins Bcl2 and Bcl‐XL were increased in cells transfected with Prom2 (Figure 6e). Since cell survival and senescence are strongly regulated by autophagy, an important recycling mechanism of damaged components in the aging heart (Mialet‐Perez & Vindis, 2017) we sought to determine whether Prom2 expression could regulate autophagic flux. While basal levels of LC3II and p62 were not modified by Prom2 overexpression, we observed an inhibition of the autophagic flux in the presence of Bafilomycin A1 (BafA1), an inhibitor of the lysosomal V‐ATPase (Figure 6f) suggesting that Prom2 could inhibit the synthesis or maturation of autophagosomes. Consistently, Prom2 increased phosphorylation of the mTOR substrate p70S6K (Figure 6g), indicative of mTOR activation, a main negative regulator of autophagic flux (Saxton & Sabatini, 2017).

### 3.5 Prom2 downregulation mitigates SIPS

**FIGURE 7:**
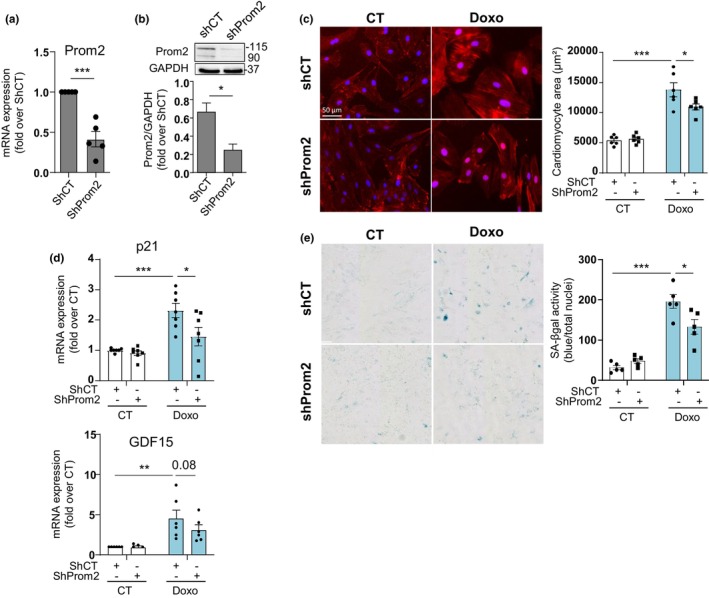
Prom2 silencing mitigates SIPS. (a) The mRNA expression of Prom2 in H9C2 cells transfected with ShCT or ShProm2 for 72 h. Data are expressed as mean ± SEM of *N* = 5 experiments. (b) Protein expression of Prom2 in H9C2 cells transfected with ShCT or ShProm2 for 6 days. Data are expressed as mean ± SEM of *N* = 3. (c) Immunofluorescence staining of WGA in H9C2 cells transfected with ShCT or ShProm2 for 72 h and then treated with Doxo for an additional 72 h. Scale bar = 50 μm. Data are expressed as mean ± SEM (*N* = 6). (d) p21 and GDF15 mRNA expression in H9C2 cells transfected with ShCT or ShProm2 for 24 h and treated with Doxo for an additional 72 h. Data are expressed as mean ± SEM (*N* = 7). (e) SA‐βgal activity in H9C2 cells transfected with ShCT or ShProm2 for 24 h and treated with Doxo for 72 h. The area of blue, indicative of SA‐βgal^+^ cells, divided by the total number of nuclei is displayed in the graph and expressed as mean ± SEM (*N* = 5).

Finally, to assess the potential of Prom2 inhibition as a novel strategy to hinder senescence‐related alterations in cardiac cells, we silenced Prom2 expression with shRNA and performed Doxo treatment. At baseline, shProm2‐transfected cells showed downregulation of Prom2 mRNA and protein levels by about 60% (Figure 7a,b). Most interestingly, Prom2 silencing reduced Doxo‐induced hypertrophy and activation of p21 gene expression in H9C2 cells compared to shCT‐transfected cells (Figure 7c,d). The SASP factor GDF15 was also decreased, although not significantly (Figure 7d). In addition, the late marker of senescence SA‐βgal was also significantly reduced in shProm2‐transfected cells (Figure 7e). These results indicate that Prom2 takes part of the Doxo‐induced senescence program in cardiac cells.

## 4 DISCUSSION

Here, by studying the transcriptome of isolated cardiomyocytes, we identified for the first time the transmembrane glycoprotein Prom2 as the most upregulated gene in aged mice compared to young mice. We found that age‐induced upregulation of Prom2 was specific for the heart compared to other tissues, and was also observed in atrial tissues of patients with HFpEF, a frequent disease in the elderly. In aged hearts or in different models of SIPS, Prom2 expression was driven by a p53‐dependent signaling pathway and was associated with cardiomyocyte hypertrophy. Finally, we showed that overexpression of Prom2 was sufficient to drive senescence and SASP in vitro, while Prom2 silencing mitigated Doxo‐induced senescence.

Senescence pathways may vary according to organs and cell types so there is a need to identify cell‐specific molecular signatures (Bernardes de Jesus & Blasco, 2012; Sharpless & Sherr, 2015). While the role of senescence in the heart has been recently uncovered, translation into relevant human applications is limited by our fragmentary understanding of the basic biology of the different populations of senescent cardiac cells (Mehdizadeh et al., 2022; Owens et al., 2021). In‐depth understanding of how specific signaling pathways promote pathological cardiac aging and HFpEF requires the development of new predictor tools to detect and follow cardiomyocyte senescence. In this study, we characterized a network of genes (Prom2, Pah, KcnK1, Cyp2b10) specifically upregulated in aged mouse cardiomyocytes with strong enrichment in cardiac tissues compared to other organs. Cyp2b10 belongs to the large family of cytochrome P450 (CYP) enzymes that are primarily involved in the biotransformation of drugs and xenobiotics in the liver (Zanger & Klein, 2013). Cyp2b10 is only present in rodents and the orthologous gene is Cyp2b6 in humans and primates. The high variability of expression of Cyp2b6 among individuals due to genetic polymorphisms and gene‐induction by many drugs, makes it difficult to use it as a reliable marker. Interestingly, the two other genes, Kcnk1 and Pah, have previously been shown to be up‐regulated in mouse cardiac aging from FBV mice using microarrays (Inuzuka et al., 2009) and more recently in the *tabula muris* single cell transcriptomic (Tabula Muris, 2020). However, in the rat, we observed opposite regulation of the potassium channel Kcnk1 with aging, meaning that the increased expression might be restricted to the mouse. From a functional point of view, the increase in Pah expression with cardiac aging is consistent with a recent study performed by Czibik et al. (2021). The authors observed that aged mice had increased circulating levels of phenylalanine, leading to ectopic expression of its metabolizing enzyme PAH in the heart. Additionally, they found that phenylalanine contributed to senescence, fibrosis and ventricular dysfunction as administration of phenylalanine in young mice accelerated cardiac aging (Czibik et al., 2021). At present, the translational relevance of these findings will necessitate further exploration since we were unable to detect Pah mRNA expression in the human atrial samples. On the other hand, we found that upregulation of Prom2 with cardiac aging was well conserved across different species such as Mouse, Rat and Human and occurred in both males and females. Since then, our observations have been confirmed in the *Tabula Muris*, which provided additional indications that Prom2 was one the most upregulated genes in the heart as soon as 9 months in mice and at older ages (Figure S3a–c) (Tabula Muris, 2020). Remarkably, a trajectory of linear increase with aging in both males and females signifies that Prom2 expression level could be a novel marker of cardiac aging (Figures S4a and S5a,b). The *Tabula Muris* data also confirmed the absence of age‐regulation of Prom2 in other tissues, except in the brain, which follows comparable increase as found in the heart (Figure 4b).

The identification of Prom2 as the most upregulated gene in aged cardiomyocytes is intriguing since it was mainly described in renal epithelial cells, digestive tract and other epithelial tissues (Fargeas et al., 2003). Up to date, the role of Prom2 in the heart has never been evaluated. This is not really surprising since our data show that its expression level is particularly low in young hearts and becomes significant in old hearts, which supports a particular role in aging. Prom2 is a pentaspan membrane glycoprotein showing partial homology (25%–30%) with the stem cell marker Prominin‐1 (CD133) (Fargeas et al., 2003) that binds directly to cholesterol through a cholesterol‐binding domain in its C‐term protein sequence (Florek et al., 2007). Interestingly, Prom2 has a high degree of interspecies conservation with 73% homology between human and mouse (Fargeas et al., 2003). In epithelial cells, Prom2 has been localized in cell protrusions and overexpression of Prom2 decreased the number of caveolae and increased the phosphorylation of caveolin1 (Singh et al., 2013). Caveolae are membrane microdomains mainly composed of cholesterol and specific proteins known as caveolins (Caveolin‐1, ‐2, or ‐3), which serve as transduction platforms for signaling pathways (Lamaze et al., 2017). Dowland et al. showed that Prom2 disrupted caveolae formation by sequestering membrane cholesterol in uterine epithelial cells (Dowland et al., 2018). With aging, a loss of caveolae structures is observed in cardiac tissue (Ratajczak et al., 2003). In addition, caveolae negatively regulate cardiomyocyte hypertrophy in vivo and in vitro (Horikawa et al., 2011; Mialet‐Perez et al., 2012). This is consistent with our findings that overexpression of Prom2 in cardiomyoblasts promotes hypertrophy. However, the role of caveolae in this context will necessitate further studies. Most interestingly, we report for the first time that overexpression of Prom2 is sufficient to drive senescence in cardiomyoblasts, characterized by the activation of p21 and p15/p16 pathways together with SA‐βgal staining and increase in SASP factors GDF15 and IL6. This phenotype is associated with a strong activation of the pro‐senescence mTOR pathway and the inhibition of autophagic flux. Autophagy is altered during cardiac aging with a downregulation of many autophagic proteins and profound consequences on heart homeostasis (Tabibzadeh, 2023), and knockdown of Atg5 in young mice recapitulates the features of cardiac aging (Taneike et al., 2010). Since autophagy is generally described as an anti‐senescence process, its inhibition by Prom2 could further reinforce the senescence of cardiomyoblasts.

In more recent years, Prom2 has been identified as a new marker of cancer cells, conferring tumorigenicity and resistance to chemotherapy (Saha et al., 2020). Most interestingly, using RNAseq approaches, upregulation of Prom2 has been recognized as a strong survival response against ferroptosis in mammary epithelial and breast carcinoma cells (Brown et al., 2019). Mechanistically, Prom2 promoted the formation of multivesicular bodies (MVBs) and exosomes that transported iron out of the cell, thereby inhibiting ferroptosis. Interestingly, ferroptosis has been shown to mediate the cardiotoxic effect of dox treatment through accumulation of mitochondrial Fe^2+^ and lipid peroxidation in cardiomyocytes (Abe et al., 2022). In this context, Prom2, by increasing iron efflux and promoting ferroptosis‐resistance, could favor instead dox‐induced senescence. In our study, we observed that overexpression of Prom2 in cardiomyoblasts enhanced resistance to acute cell death and upregulated two cell survival factors Bcl2 and Bcl‐XL, which are the main targets of the senolytic navitoclax. Prom2 being part of the senescent cardiomyocyte survival mechanism opens the possibility to selectively remove these cells in pathological or aged hearts. Only very few studies have tried to eliminate senescence in a cell‐specific manner. In a very recent report, an osteocyte‐selective conditional senolysis approach using DMP1‐cre mice crossed with p16‐LOX‐ATTAC mice was compared to global senolysis with the *p16‐INK‐ATTAC* mice (Farr et al., 2023). Clearance of senescent osteocytes gave lower improvement in bone integrity and only a limited effect on the SASP, compared to systemic senolysis. Thus, the effect of local senolysis might be impaired by the persistence of SASP factors released by other senescent cells and acting at distance (Farr et al., 2023). Nevertheless, this study underscores the need of understanding organ‐level cell‐specific senescence and highlights the potential relevance of Prom2 in this context.

Concerning the regulatory mechanisms of Prom2 transcription, the activation of p38MAP kinase has been implicated in the phosphorylation and activation of the Heat Shock transcription factor HSF1 to drive Prom2 upregulation in cancer cells (Brown et al., 2021). In the present study, we identified another mechanism of regulation of Prom2 expression. Considering that Prom2 promoter exhibited a p53‐responsive element we investigated the role of p53 by using Nutlin‐3a. With this treatment, we observed specific activation of the p53/p21 pathway and upregulation of Prom2 without activation of the INK4 family proteins (p15, p16).

Most interestingly, we found that Prom2 was not only increased in aged hearts, but also in different models of SIPS, indicating that this determinant could be shared between physiological aging and age‐associated disease. For instance, thanks to the analysis of atrial samples from patients, we reported that Prom2 increased in aged human samples with HFpEF, suggesting a causative link between Prom2 expression and senescence‐associated cardiac disease. HFpEF is the most frequent form of HF in elderly or obese patients (Teramoto et al., 2022). Thus, Prom2 could serve as a new marker of HFpEF and also as a new target for senolysis in the heart.

In conclusion, we have identified Prom2 as a novel marker of cardiomyocyte senescence that promotes DNA damage, senescence, SASP, hypertrophy and resistance to cell death. The identification of this new marker provides new and more fundamental notions on the general biology and function of cardiomyocyte senescence. This could give new perspectives for the tracking of senescent cardiomyocytes in vivo in future studies with senolytic or senomorphic compounds. It could also allow therapeutic targeting of senescent cardiomyocytes through new pharmacological approaches directed against Prom2 signaling pathways.

### 4.1 Limitations of the study

Although prom2 up‐regulation in cardiac samples has been validated with aging by cross‐species study, the role of prom2 in cardiac aging in vivo remains to be further explored. While we confirmed the upregulation of Prom2 in aging hearts of female mice, the validation in female rats will need further investigation. In human study, a number of confounding factors could modulate the results of the analysis such as medications or the presence of hypertension and diabetes, which are very frequent in elderly subjects. In order to circumvent these limitations, a larger cohort of samples will be required to corroborate these findings. In the present study, the mechanistic role of prom2 was conducted in vitro using H9C2 in the early steps of SISP in response to diverse stressors (Doxo, MitoROS, telomeres damage). Despite their expression of the cardiac marker α‐actinin, H9C2 display some major phenotypic differences with post‐mitotic cardiomyocytes, as they are replicative cells and derive from embryonic heart tissue. However, these cells reproduce important features of the early steps of cardiomyocyte senescence such as hypertrophy, SA‐βgal activity, p53 dependent cell cycle inhibition and SASP factors, as previously described (Manzella et al., 2018).

## AUTHOR CONTRIBUTIONS

DM, YS, and KF designed and performed the experiments, analyzed data and participated in paper writing. ED, HM, SB, and GC performed some experiments and analyzed the corresponding data. SB, GC, and OL obtained patient's approval, generated the human atrial bank and provided the samples. AP and FL helped to design the research and reviewed the manuscript. JMP and VDE designed and supervised the whole study, analyzed data and wrote the paper.

## FUNDING INFORMATION

This work was supported by grants from the Institut National de la Santé et de la Recherche Médicale (INSERM), Agence Nationale de la Recherche (ANR‐19‐CE14‐0038‐01), Région Occitanie, Fédération Française de Cardiologie, Fondation pour la Recherche Médicale (“Equipes FRM 2021, EQU202103012601”), Région Pays de la Loire. KF is recipient of a postdoctoral fellowship from Lefoulon‐Delalande foundation.

## CONFLICT OF INTEREST STATEMENT

No conflict of interest.

## Supporting information


Data S1:

## Data Availability

The data that support the findings of this study are available from the corresponding authors upon reasonable request.
