# Supplementary material for: Identification of Prominin‐2 as a new player of cardiomyocyte senescence in the aging heart
Source: Aging Cell. 2024 May 17;23(9):e14204. doi: 10.1111/acel.14204 (PMC11488343; doi:10.1111/acel.14204)
Supplement: Supplementary file 1 — Data S1: [file ACEL-23-e14204-s001.docx]

**Supplemental materials and methods**

***In vitro* stress-induced premature senescence models**

For neonatal rat ventricular myocytes (NRVMs), neonatal rats of 1–2 days old were euthanized, and hearts were excised before the atria were removed. Primary culture of NRVMs was subsequently performed as previously described (Santin et al. 2016). NRVMs were transiently transfected with either TRF1-FokI-D450A or TRF1-FokI plasmids using Lipofectamine 2000 (Thermofisher) at a ratio of 3μL Lipofectamine 2000 to 1μg DNA following the manufacturer’s protocol and collected after 5 days. For MAO-A activation, H9C2 were treated with MAO substrate t

yramine (500 µM) for 96 h.

**Female mice cohort**

Female mice in the C57BL/6-SV129 background were housed in a pathogen-free facility until the age of 3 months (N=6) or 24 months (N=10). All animal experiments were approved by the Animal Care and Use Committees of the University of Toulouse.

**Supp References**

Santin Y, Sicard P, Vigneron F, Guilbeau-Frugier C, Dutaur M, Lairez O, Couderc B, Manni D, Korolchuk VI, Lezoualc'h F, Parini A, Mialet-Perez J (2016) Oxidative Stress by Monoamine Oxidase-A Impairs Transcription Factor EB Activation and Autophagosome Clearance, Leading to Cardiomyocyte Necrosis and Heart Failure. Antioxid Redox Signal 25 (1):10-27. doi:10.1089/ars.2015.6522
